# *NR4A3* and *CCL20* clusters dominate the genetic networks in CD146^+^ blood cells during acute myocardial infarction in humans

**DOI:** 10.1186/s40001-021-00586-8

**Published:** 2021-09-26

**Authors:** Yan-hui Wang, Chen-xin Li, Jessica M. Stephenson, Sean P. Marrelli, Yan-ming Kou, Da-zhi Meng, Ting Wu

**Affiliations:** 1 College of Mathematics and Systems Science, Shandong University of Science and Technology, 579 Qianwangang Road, Huangdao District, Qingdao, 266590 Shandong China; 2 Department of Neurology, University of Texas Health Science Center at Houston, 6431 Fannin street, Houston, TX 77031 USA; 3 College of Applied Science, Beijing University of Technology, 100 Pingleyuan, Beijing, 10024 Chaoyang China

**Keywords:** Acute myocardial infarction (AMI), CD146, Pearson network, Clustering coefficient, Differential connectivity genes (DCGs)

## Abstract

**Background:**

CD146 is a tight junction-associated molecule involved in maintaining endothelial barrier, and balancing immune–inflammation response, in cardiovascular disease. Notably, peripheral CD146^+^ cells significantly upsurge under vessel dyshomeostasis such as acute myocardial injury (AMI), appearing to be a promising therapeutic target. In this study, with a new view of gene correlation, we aim at deciphering the complex underlying mechanism of CD146^+^ cells’ impact in the development of AMI.

**Methods:**

Transcription dataset GSE 66,360 of CD146^+^ blood cells from clinical subjects was downloaded from NCBI. Pearson networks were constructed and the clustering coefficients were calculated to disclose the differential connectivity genes (DCGs). Analysis of gene connectivity and gene expression were performed to reveal the hub genes and hub gene clusters followed by gene enrichment analysis.

**Results and conclusions:**

Among the total 23,520 genes, 27 genes out of 126 differential expression genes were identified as DCGs. These DCGs were found in the periphery of the networks under normal condition, but transferred to the functional center after AMI. Moreover, it was revealed that DCGs spontaneously crowded together into two functional models, *CCL20* cluster and *NR4A3* cluster, influencing the CD146-mediated signaling pathways during the pathology of AMI for the first time.

**Supplementary Information:**

The online version contains supplementary material available at 10.1186/s40001-021-00586-8.

## Introduction

Cluster of differentiation 146 (CD146)/melanoma cell-associated molecule is an essential immunoglobulin-like protein initially discovered in metastatic melanoma [1]. It locates at endothelial tight junctions across all vessel beds, mediating physiological and pathological events under vascular dyshomeostasis [2, 3]. Pioneering researchers regard CD146 as a historical marker for isolating circulating endothelial cells that slough off inflamed vasculature [4]. Over several decades, CD146 has also been discovered in other cell types including mesenchymal stem cells [5], endothelial progenitor cells [6], macrophages [7], T helper 17 cells [8], B lymphocytes [9], T lymphocytes [9, 10], and natural killer cells [9]. The CD146^+^ circulating cells occupy about 2% of peripheral mononuclear cells in healthy individuals [9] and most notably, this percentage increases in certain conditions associated with vascular dysfunction like myocardial infarction, connective tissue diseases, and cancers [6, 11–13]. Moreover, CD146-activated T cells have shown an enhanced ability to interact with endothelium in adhesion, rolling, and transmigration, evidenced by human and murine studies [14, 15]. Given its multiple functions in vessel structure, angiogenesis, and lymphocyte activation and its enabled detection in the bloodstream, CD146 appears to be a potential target for vascular disorders [16–18].

Complex networks are of great interest to researchers in the fields of computational biology and bioinformatics [19–21]. It has been gradually extended from initial gene comparison to protein–protein network modeling, to protein–genetic investigation, and up to the disease–disease association exploration [22]. Most of the successful bioinformatics approaches that identify the initial key genes, however, have been based on only gene expression comparison and restrict the following analysis to the top differential expression genes (DEGs) without paying attention to the gene interaction rearrangement [23, 24]. Instead, the hub-structured network is an important motif that is, to our best knowledge, leading the genome-wide association characterization in complex networks [25, 26]. It generates the structure view angle to present the innermost gene–gene interaction, giving a comprehensive understanding of the underlying mechanisms of disorders.

Acute myocardial injury (AMI) dataset GSE 66,360 was focused on the performance of CD146^+^ populations during early AMI development [12, 27–29]. In this paper, we try to decipher gene reorientation within the correlation network structure parameter analysis [30, 31], extract the optimal gene collections which are termed the differential connectivity genes (DCGs), and reveal functional gene clusters which likely lead to the pathogenesis of peripheral CD146^+^ blood cells during the development of AMI in human for the first time.

## Materials and methods

### Data

The GSE66360 [12] gene transcription profile data of human AMI in the NCBI database was selected as the primary interest. Clinical subjects including 50 healthy individuals and 49 AMI patient subjects were recruited in the original investment by the Topol group. To gather the data, CD146^+^ cells were obtained by CD146-based magnetic immunoisolation from the subjects’ blood samples. RNA samples were isolated from the CD146^+^ cells and processed by Affymetrix human U133 Plus 2.0 array. In this study, two cohorts were formed: a discovery cohort, consisting of 22 healthy subjects (control group) and 21 AMI patients (AMI group), which were used for the discovery of genes and appropriate testing methods, and a validation cohort, consisting of 28 healthy subjects and 28 AMI patients, which were used for the validation of the genes and methods discovered in the discovery cohort. No data were excluded from the original databases used during this study.

### Study design

**Fig. 1:**
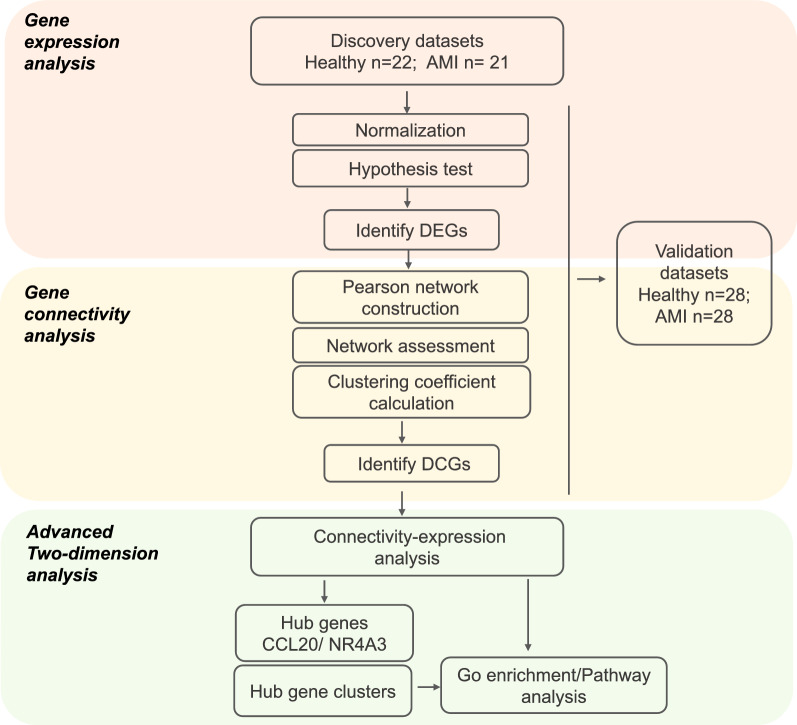
Flowchart for study design. *DEGs* differential expression genes; *DCGs* differential connectivity genes

First, using the hypothesis test published by our group [33], we distinguished DEGs based on the gene expression profile in the discovery cohort and then verified in the validation cohort. Second, the gene networks of DEGs were constructed based on the Pearson coefficients, followed by the network separation assessment. Third, the clustering coefficient, which is a parameter indicating gene connectivity, was calculated for each DEG under each gene network [32]. Accordingly, genes with a clustering coefficient that represented a consistent increase in the AMI group among different cohorts were labeled as DCGs. Finally, two-dimensional analysis of gene connectivity and expression was employed, hub gene clusters were identified, and gene enrichment analysis was performed (Fig. 1).

### Identify DEGs

A total of 23,520 genes were screened in each sample. The hypothesis test was used to screen the DEGs between control and AMI group [33]. The method primarily gave weight for the distribution shape of the expression spectrum. If the distribution shape was different between the two groups, then the gene expression was different with regard to significance level *α*_1_. If not, a normal distribution test (significance level *α*_2_) and homogeneity test of variance (significance level *α*_3_) would be carried out. *T *test or Welch’s *t *test (significance level *α*_4_) was used for normal distribution; rank sum test (significance level *α*_4_) was used for non-normal distribution with a similar distribution of expression spectrum [33]. We defined *α*_1_  =  0.00001, *α*_2_  =  0.00002, *α*_3_  =  0.00001, and *α*_4_  =  0.00001 as the significance levels of the hypothesis tests for DEGs selection regarding the limited input gene size of the following connectivity analysis.

### Clustering coefficient

A local clustering coefficient was introduced to measure the compactness, or the connectivity of genes within a suspected cluster, of a complete array formed by the adjacent nodes within a network [34]. To clarify, assume that a node *i* in a network was connected to *k*_*i*_ nodes. The *k*_*i*_ nodes were called neighbors of node *i*. The ratio of the actual number of edges *E*_*i*_ and the total number of possible edges *k*_*i*_(*k*_*i*_ − 1)/2 between *k*_*i*_ nodes were defined as the clustering coefficient, *C*_*i*_, of node *i*; that is, *C*_*i*_  =  2*E*_*i*_/[*k*_*i*_(*k*_*i*_ − 1)].

### Pearson network construction and assessment

Pearson correlation networks of DEGs were constructed according to the absolute value of Pearson coefficients. Two genes were considered correlated if the absolute value of the Pearson coefficient was greater than the threshold *x* (0  <  *x*  <  1), at which point a line could be drawn between the two genes. In the cases when genes were not correlated, there would be no link in the network and thus no line would be drawn. Gene clusters were determined by examining the clustering coefficients, and those with a non-zero value could be labeled clusters. Gene clusters represent functional modules as a whole with varying degrees and connectivity; while the degree describes the number of genes connected to one another. The average clustering coefficients of DEGs were calculated to evaluate the overall separation of the control and AMI networks. This analysis was performed in R i386 3.6.2.

Natural biological networks are scale-free networks and the degree distributions follow the power-law exponential distribution index range 2–3 [35, 36]. We specifically looked at the gene networks under threshold 0.5 and 0.7 because the power-law indexes of degree distribution in discovery cohort were in the range of 2–3. We then presented the corresponding networks in discovery cohort and validation cohort of this study (Additional file 2: Table S1).

### Identify DCGs

In the analysis of network connection parameters, the greater the difference between the control and AMI group, the higher the correlation with AMI. The following describes our unique identifying method. Assume that the average clustering coefficient of the control group and AMI group could be separated at the threshold (0.1, 0.9). First, the clustering coefficients of each gene in the Pearson correlation networks under the threshold 0.1–0.9 of the control group and AMI group were calculated with step length 0.1. Secondly, the average clustering coefficients of each gene through threshold 0.4–0.8 were calculated to compare changes in connectivity between the two groups within the validation and discovery cohorts. Finally, if clustering coefficient differences in discovery cohort and validation cohort were consistently greater than 0.1 between the AMI group and the control group, the genes were identified as candidates for DCGs.

To test the reliability of the proposed candidate genes across different datasets, we expanded our method to a combination cohort, which included all subjects in both discovery and validation cohorts. This increased the number of subjects, but also introduced some variation in the data due to the less categorized subject population. The overall network between the control and the AMI groups were still separable through threshold 0.4–0.8 (data not shown). The 27 out of 39 candidate genes that still showed clustering coefficient differences greater than 0.1 in this combination cohort were defined as DCGs.

### Gene set enrichment

Gene set enrichment was performed by the STRING server. Biological process, reactome pathways, and protein–protein association networks were generated for *CCL20* cluster, *NR4A3* cluster, and DCGs.

### Graphs

Heatmaps of DEGs and DCGs were generated by using heatmap.2 function in the gplots package. Networks were computed by using igraph::graph.data.frame function. Layout algorithm of layout.kamada.kawai was used for visualizing the overall DEGs networks and the connections for individual genes. Layout algorithm of layout.circle was used to visualize the gene connections within DEGs and DCGs in circle view. Cytoscape network function was used to generate the clustered DCGs networks.

## Results

### DEGs identification

**Fig. 2:**
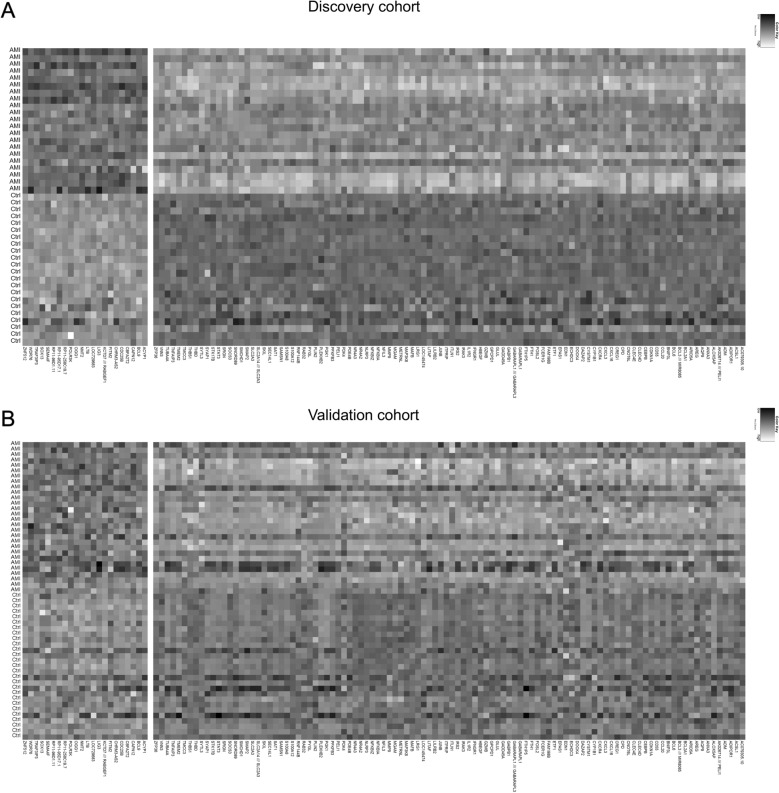
Gene expression profiles of DEGs. 126 genes show significant differential expressions between the AMI and the control groups in the discovery cohort (**A**) and validation cohort (**B**), defined as DEGs. *DEGs* differential expression genes

In our initial analysis, 126 out of 23,520 genes’ expression levels were significantly altered in the AMI group compared to the control group in discovery cohort, defined as DEGs, with the majority (79 of 126) demonstrated an upregulation feature (Fig. 2A). The validation cohort showed a similar expression pattern (Fig. 2B).

### Assessment of DEGs’ networks

**Fig. 3:**
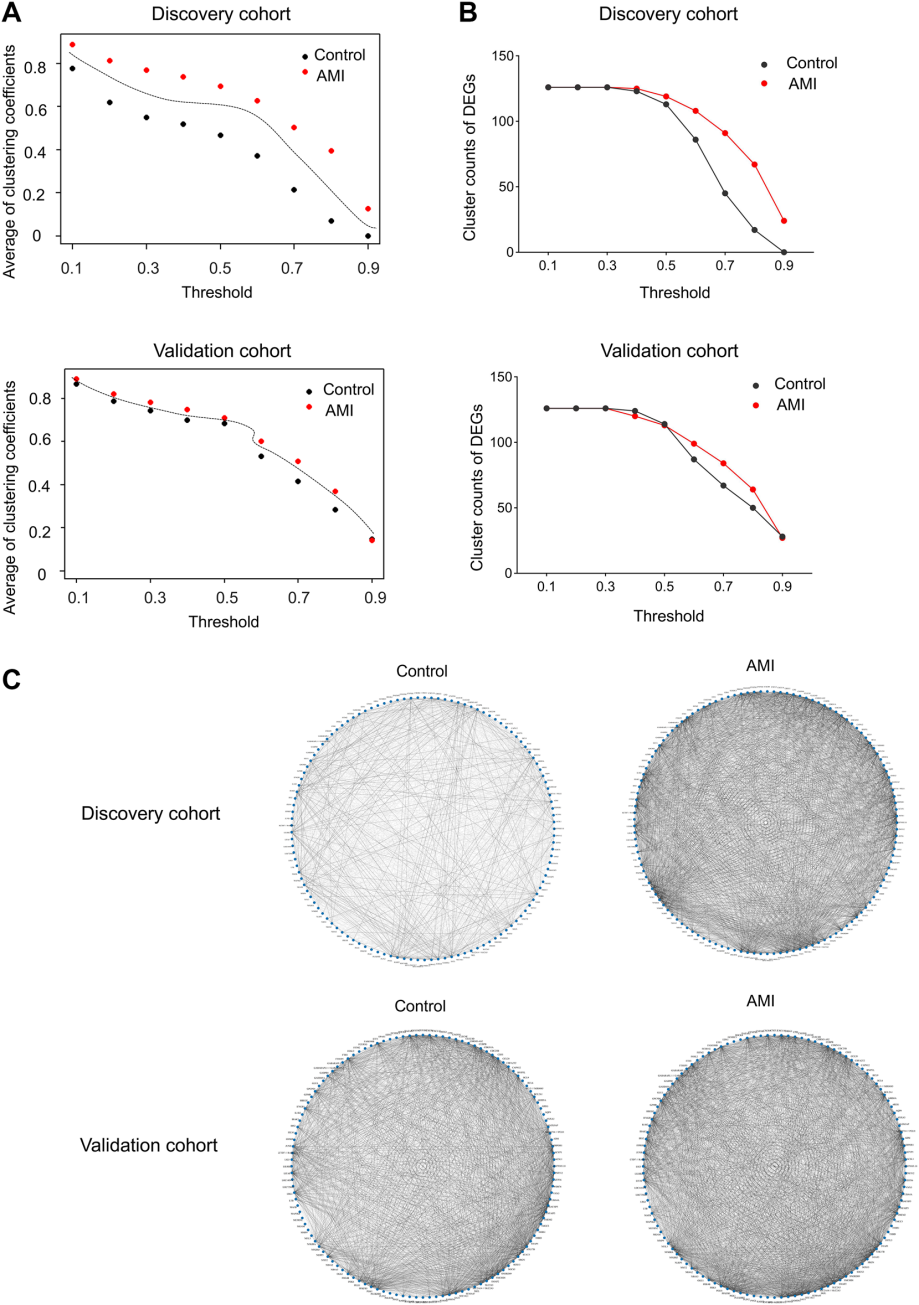
Assessment of DEGs’ networks. Networks in the control and AMI groups are independent and separable according to the average clustering coefficients of DEGs (**A**). Number of clusters within DEGs’ networks progressively decline when thresholds increase from 0.1 to 0.9 (**B**). The AMI group has a lower slope decline. The gene networks of DEGs in the AMI group have more complex connection, compared to that in the control group (**C**). Networks are present under threshold 0.5 and 0.7. Darker line represents connections under threshold 0.7; lighter line represents connections under threshold 0.5. *DEGs* differential expression genes

The overall gene networks of DEGs in the control group and the AMI group were distinctly independent through a large range of thresholds in discovery cohort and validation cohort (Fig. 3A). The networks in discovery cohort were separable through threshold 0.1–0.9 and in validation cohort were separable through 0.1–0.8. The average separable widths of the discovery cohort and validation cohort were 0.218 and 0.0518, respectively. The validation cohort showed a narrower split range possibly due to the variations between two cohorts; for instance, the differential sample size, age, and co-morbid disorders.

In addition, the gene connections within DEGs’ networks in the AMI group were more complex than those in the control group in both cohorts (Fig. 3C). In the discovery cohort, the number of gene clusters within AMI network gradually decreased from 125 to 67, when threshold increased from 0.4 to 0.8, while it more sharply decreased from 123 to 17 in the control group (Fig. 3B). Similarly, the clusters declined with a lower slope in the AMI group compared to the control group in validation cohort (Fig. 3B).

The data described above suggested that gene networks of DEGs were largely and consistently disturbed by AMI stimulation as seen in two independent cohorts, verifying our findings. Thus, DEGs and DEG-networks were mathematically reliable and hereafter could be set as the foundation for in-depth gene interaction data mining.

### DCGs identification and connectivity analysis

**Fig. 4:**
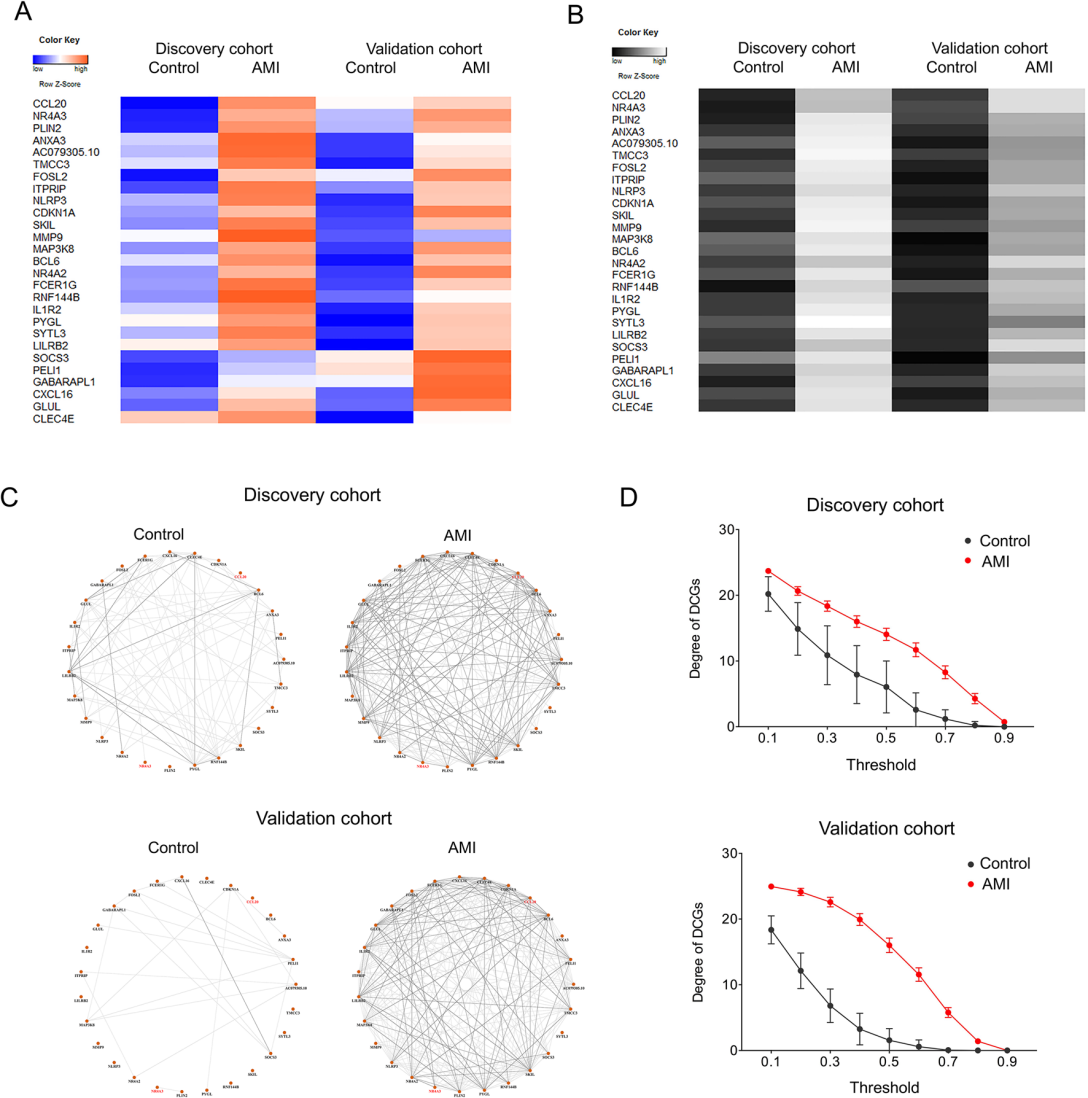
Identification of DCGs. Genes for which the clustering coefficient increased by over 0.1 in the AMI group in both discovery cohort and validation cohort, are revealed as DCGs (**A**). Gene expression profile of DCGs shows a stable increase in the AMI group in both cohorts (**B**). The connections among DCGs in the AMI group are denser (**C**) and the average degrees of DCGs in AMI group are higher (**D**) compared to the control group in both cohorts. Networks are presented under threshold 0.5 and 0.7. Darker lines represent connections under threshold 0.7; lighter line represents connections under threshold 0.5. Degrees are presented as mean  ±  SEM. *DCGs* differential connectivity genes

Beyond DEGs, we identified 27 genes as DCGs whose clustering coefficient difference was greater than 0.1 in the discovery cohort, the validation cohort, and the extended combination cohort (Fig. 4A; Additional file 2: Table S2). The sub-networks of DCGs presented obvious tighter connections in the AMI group compared to the control group, in both discovery and validation cohorts (Fig. 4C). When threshold increased from 0.4 to 0.8, the average degree of DCGs progressively decreased from 16.0 to 4.30 in the AMI group, while it decreased from 7.93 to 0 in the control group in discovery cohort (Fig. 4D). Similarly, in the validation cohort, this number decreased from 19.9 to 1.41 in the AMI group while from 3.26 to 0 in the control group (Fig. 4D). In addition to the clustering coefficients, gene expression of those DCGs showed a steady increase in the AMI group in both the discovery and validation cohorts (Fig. 4B).

**Fig. 5:**
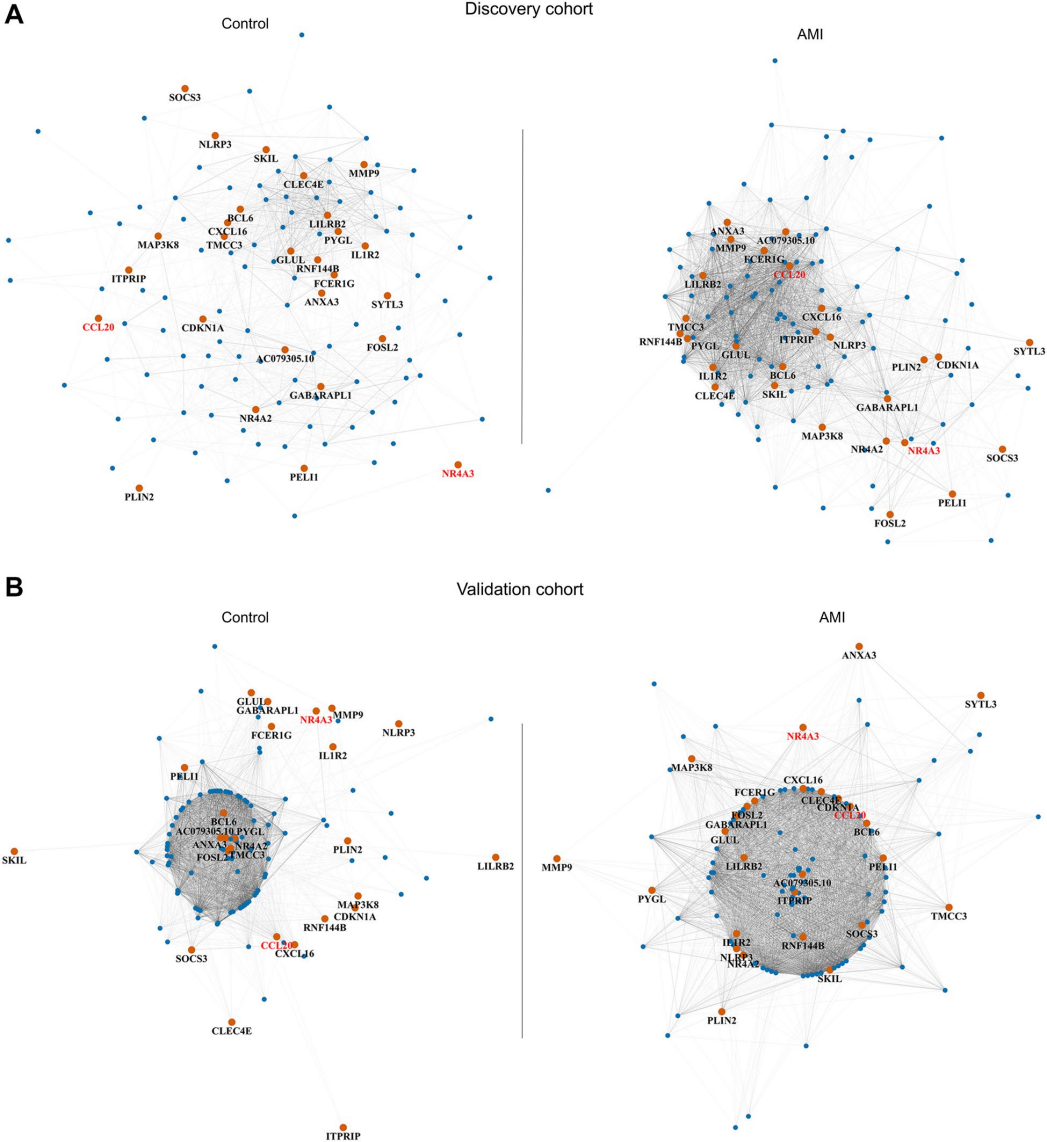
Visualization of DCGs in DEGs’ networks. The networks of DEGs in discovery cohort (**A**) and in the validation cohort (**B**) indicate that the DCGs participate in distinctive ways in the control group and in the AMI group. DCGs switch to central functional position of networks and participate in more intricate connections under AMI situation. Yellow nodes indicate the DCGs. Red gene names indicate the hub genes. *DEGs* differential expression genes; *DCGs* differential connectivity genes

Therefore, we proposed that the networks’ differential of all DEGs was largely attributed to the connection changes within DCGs. As visualized in kamada-kawai layout, the DCGs randomly participated in the DEGs’ network and connected to a few genes under a normal steady state. However, they appeared to interact with more functional genes and shift into central positions of the clusters after AMI in both discovery and validation cohorts (Fig. 5).

### Two-dimensional analysis of gene connectivity and gene expression

**Fig. 6:**
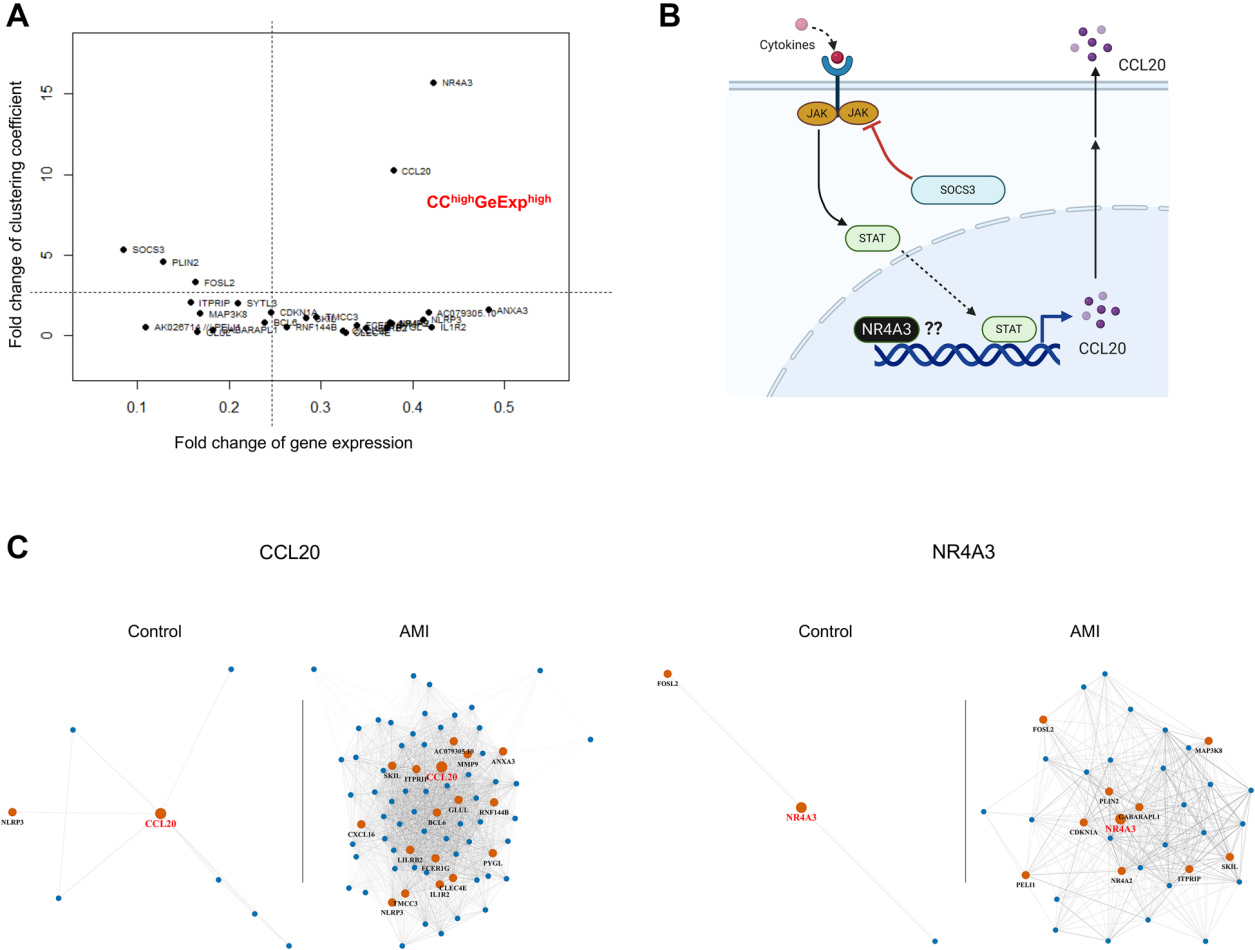
Analysis of gene connection and expression of DCGs in discovery cohort. The analysis of clustering coefficient and gene expression revealed *CCL20* and *NR4A3* as hub genes (**A**). The *CCL20* is a chemoattractant while *NR4A3* is a nuclear factor receptor (**B**). Subgraphs of *CCL20* and *NR4A3* substantiate their important roles in AMI development (**C**). Networks are presented under threshold 0.5 and 0.7. Darker lines represent connections under threshold 0.7; lighter lines represent connections under threshold 0.5. *DCGs* differential connectivity genes; *CC* clustering coefficient; *GeExp* gene expression

As the power-law indexes of degree distribution in the discovery were in the range of natural networks, we regarded the discovery cohort as a more precise dataset and it was selected for the following analysis. The average clustering coefficient and gene expression of DCGs were plotted for two-dimensional analysis (Fig. 6A). *NR4A3* and *CCL20* presented high levels of clustering coefficient and gene expression changes, defined as CC^high^GeExp^high^ genes. *SOCS3*, *FOSL2*, *PLIN2* were the genes that were found to have high clustering coefficient changes (fold-change  >  2) with low expression changes, defined as CC^high^GeExp^low^ genes; while *IL1R2*, *NLRP3*, *ANXA3* and *AC079305.10* were the genes that were found to have high expression changes (fold-change  >  0.4) with low clustering coefficient changes, defined as CC^low^GeExp^high^ genes. All information on these genes is shown in Table 1. Subgraphs of *NR4A3*, *CCL20,* and other DCGs provided evidence that supported their increased gene connectivity after AMI in the discovery cohort (Fig. 6C, Additional file 1: Figure S1A, B).

**Table 1:** Category of DCGs

Genes	Name	Clustering coefficient fold-change	Gene expression fold-change
CC^high^GeExp^high^			
*NR4A3*	Nuclear receptor subfamily 4 group A member 3	15.7	0.422
*CCL20*	C–C motif chemokine ligand 20	10.2	0.379
CC^high^GeExp^low^			
*SOCS3*	Suppressor of cytokine signaling 3	5.35	0.085
*PLIN2*	Perilipin 2	4.56	0.128
*FOSL2*	FOS like 2, AP-1 transcription factor subunit	3.30	0.163
CC^low^GeExp^high^			
*ANXA3*	Annexin A3	1.58	0.482
*AC079305.10*	Unnamed	1.40	0.417
*NLRP3*	NLR family pyrin domain containing 3	0.982	0.411
*IL1R2*	Interleukin 1 receptor type 2	0.523	0.421
*MMP9*	Matrix metallopeptidase 9	0.749	0.377
*NR4A2*	Nuclear receptor subfamily 4 group A member 2	0.767	0.375
*PYGL*	Glycogen phosphorylase L	0.498	0.372
*LILRB2*	Leukocyte immunoglobulin like receptor B2	0.432	0.349
*FCER1G*	Fc fragment of IgE receptor Ig	0.633	0.339
*CXCL16*	C–X–C motif chemokine ligand 16	0.263	0.324
*CLEC4E*	C-type lectin domain family 4 member E	0.153	0.327
CC^low^GeExp^low^			
*ITPRIP*	Inositol 1,4,5-trisphosphate receptor interacting protein	2.03	0.158
*SYTL3*	Synaptotagmin like 3	1.98	0.209
*MAP3K8*	Mitogen-activated protein kinase kinase kinase 8	1.34	0.169
*CDKN1A*	Cyclin dependent kinase inhibitor 1A	1.41	0.246
*SKIL*	SKI like proto-oncogene	1.05	0.284
*TMCC3*	Transmembrane and coiled-coil domain family 3	1.13	0.295
*BCL6*	BCL6 transcription repressor	0.818	0.239
*PELI1*	Pellino E3 ubiquitin protein ligase 1	0.489	0.109
*GABARAPL1*	GABA type A receptor associated protein like 1	0.353	0.182
*RNF144B*	Ring finger protein 144B	0.524	0.263
*GLUL*	Glutamate-ammonia ligase	0.198	0.165

*DCGs* differential connectivity genes; *CC* clustering coefficient; *GeExp* gene expression

### NR4A3 and CCL20 clusters identification

**Fig. 7:**
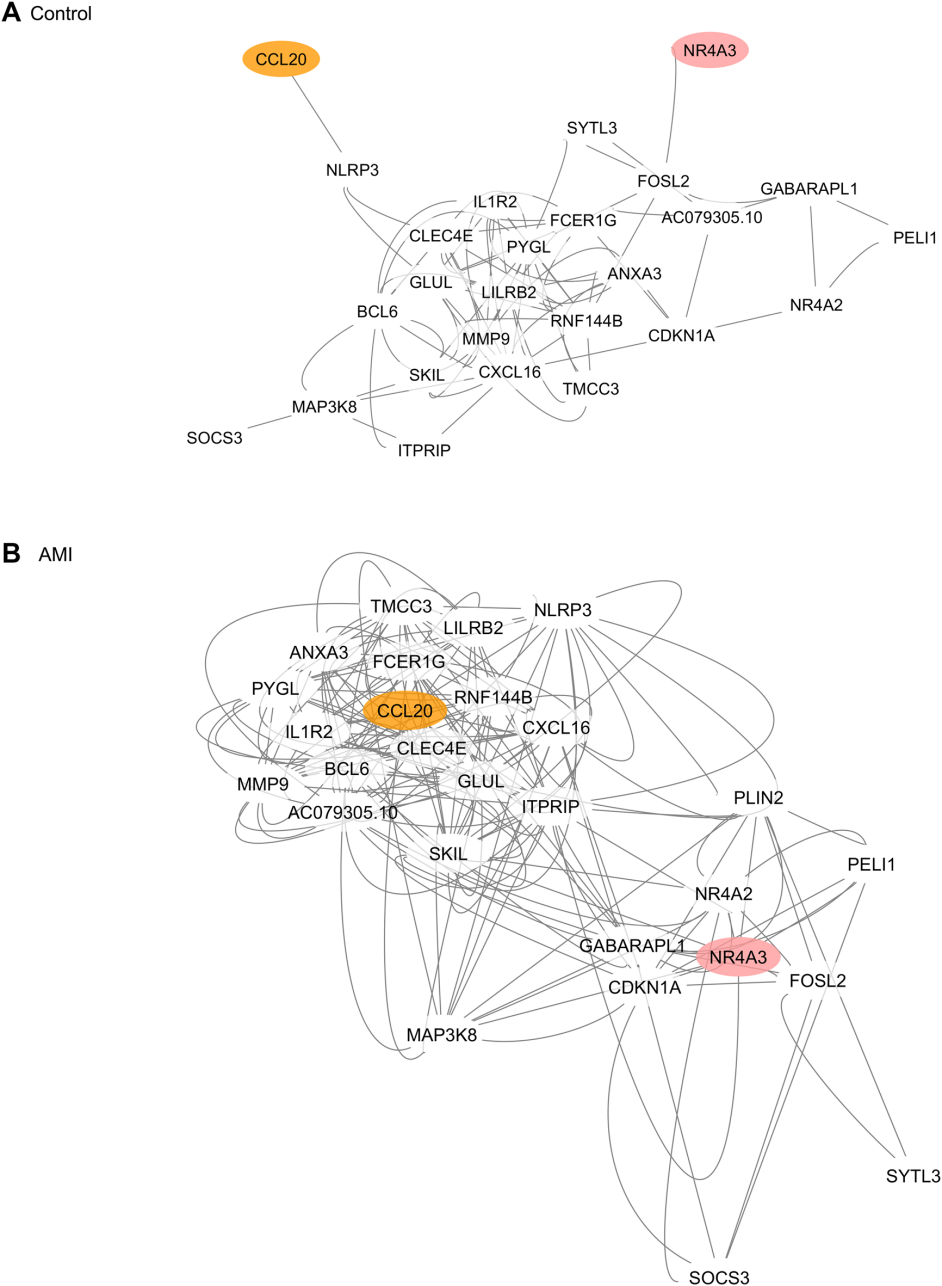
*CCL20* cluster and *NR4A3* cluster formation in early-stage AMI. *CCL20* and *NR4A3* stay in the peripheral position of DCGs’ network under normal state (**A**). However, they shift to the primary position of DCGs’ network dominating two functional clusters under AMI stimulation (**B**). *DCGs* differential connectivity genes

Taking a closer analysis of the subgraphs of individual genes, we revealed that DCGs stay “non-activated” in the control group (Fig. 7A). Interestingly, even though there were several minor clusters in the gene network, they appeared to be spontaneously gathering together as two major separate clusters after AMI stimulation (Fig. 7B, Additional file 1: Figure S1A, B). The highlighted CC^high^GeExp^high^ genes, *NR4A3* or *CCL20*, located in the center spot of each cluster, served as leading-like hub genes. *CCL20* connected with *SKIL*, *MMP9*, *ITPRIP*, *ANXA3*, *GLUL*, *CXCL16*, *IL1R2*, *TMCC3*, *NLRP3*, *PYGL*, *RNF144B*, *BCL6*, *LILRB2*, *CLEC4E*, *FCER1G*, and *AC079305.10*, identified as the *CCL20* cluster. *NR4A3* connected with *NR4A2*, *FOSL2*, *CDKN1A*, *SOCS3*, *GABARAPL1*, *ITPRIP*, *SYTL3*, *PELI1*, *MAP3K8*, and *PLIN2*, identified as the *NR4A3* cluster. While there were overlapping genes between clusters, *ITPRIP*, *SKIL* and *MAPK38* were the intermediate genes that connected both clusters according to their subgraphs (Additional file 1: Figure S1B). The clustering coefficient fold-changes of *NR4A3* and *CCL20* were 15.7 and 10.2, respectively; and the gene expression fold-changes were 0.379 and 0.422, respectively. Gene connections are partly verified by the STRING datasets (Additional file 1: Figure S2).

### Gene enrichment

Biological process analysis showed that DCGs were involved in response to organic substrates, positive regulation of leukocyte activation, immune response, immune system process, response to cytokine, and regulation of cytokine production. The *CCL20* cluster was essential to the immune response, immune system process, and regulation of localization while the *NR4A3* cluster was essential to cellular response to corticotropin-releasing hormone stimulus, positive regulation of leukocyte activation, and regulation of apoptotic process (Table 2).

**Table 2:** Top six gene enrichment outputs of DCGs and hub gene clusters

Biological process		
*CCL20* cluster	*NR4A3* cluster	DCGs
Immune response	Cellular response to corticotropin-releasing hormone stimulus	Response to organic substance
Immune system process	Positive regulation of leukocyte activation	Positive regulation of leukocyte activation
Response to organic substance	Response to organic substance	Immune response
Regulation of cytokine production	Cellular response to organic substance	Immune system process
Response to cytokine	Regulation of apoptotic process	Response to cytokine
Regulation of localization	Response to cytokine	Regulation of cytokine production
Reactome pathway		
Immune system	Signaling by interleukins	Immune system
Innate immune system	RNA polymerase II transcription	Signaling by interleukins
Signaling by interleukins	Generic transcription pathway	Interleukin-4 and interleukin-13 signaling
Cytokine signaling in immune system	Cytokine signaling in immune system	Innate immune system
Dectin-2 family	Gene expression (transcription)	Interleukin-1 signaling
Neutrophil degranulation	MyD88 cascade initiated on plasma membrane	Dectin-2 family

*DCGs* differential connectivity genes

Reactome pathway analysis revealed that DCGs were related to the immune system with regard to tasks such as signaling by interleukins, namely interleukin-1, interleukin-4 and interleukin-13, the innate immune system, and the dectin-2 family. The *CCL20* cluster was essential to immune system, innate immune system, the dectin-2 family, and neutrophil degranulation while the *NR4A3* cluster was essential to RNA Polymerase II Transcription, Generic Transcription Pathway, and MyD88 cascade initiated on plasma membrane (Table 2).

## Discussion

CD146 is a junction-associated adhesion molecule that participates in immune and inflammatory pathological processes in the initiation and development of vascular diseases [2]. CD146-activated leukocytes are recruited to the inflamed endothelium to induce the expression of chemokines and cytokines and, in doing so, progressively destroy the blood vessel barrier. Our study finds that following AMI stimulation, in CD146^+^ human blood cells, 126 out of 23,520 genes show significant differential expression (*P*  <  0.0001) and, among those, 27 genes show consistent connectivity changes and serve as DCGs. Unlike DEGs, DCGs are able to not only aggregate gene expression, but also encompass gene connectivity properties, internally coupling into functional gene clusters—*NR4A3* cluster and *CCL20* cluster—orchestrating the gene networks’ entire dynamics in CD146-associated AMI pathophysiology development. Meanwhile, *NR4A3* and *CCL20* are revealed as hub genes since they experienced both significant connectivity and expression changes after AMI stimuli. Furthermore, gene enrichment analysis shows that the DCGs are involved in inflammation–immune response, with *CCL20* being principal to the immune response and regulation of localization; while, the *NR4A3* cluster is principal to leukocyte activation, apoptotic process, and cellular response to corticotropin-releasing hormone stimulus. Such findings align with the well-known hypothesis that CD146-mediated inflammation plays an important role in the pathogenesis of AMI.

## DCGs revealed by gene connectivity network

The network structural parameter analysis method is applied to weave the gene–gene correlation network. We identify DCGs which present steadily elevated connectivity under AMI conditions in both the discovery and validation cohorts, and further confirm the upregulation seen in the combination cohort. As expected, the gene expressions of DCGs are increased after AMI, but are not distinguishable from DEGs solely by expression signatures (data not shown). *NR4A3* and *CCL20* as Queryhighlight hub genes are also defined as AMI biomarkers after pre-filtering the comorbidity-relevant genes by the original Topol group [12]. *SOCS3* tends to be the only “shared” AMI biomarker candidate revealed by other groups in which the same GSE66360 dataset is included as one of their study objects [27, 28]. Recognizing the *CCL20*, *NR4A3*, and *SOCS3* as top DCGs substantiate previous outputs and, in turn, the validity of our method is enhanced. Therefore, we recommend the gene connectivity analysis, along with gene expression signature, to be used as a powerful and unbiased way for researchers to rank the importance of candidate DEGs.

## The NR4A3 hub gene

*NR4A3* belongs to the *NR4A* orphan nuclear receptor family (with *NR4A2* and *NR4A1*), which plays an important role in AMI development. The JM Penninger group reports that *NR4A3* is the highest-ranking gene in circulating human endothelial cells under atherosclerosis [37]. Transcription analysis of human left ventricular myocardium shows that *NR4A3* upregulates during ischemia and reperfusion in normal and chronic ischemic myocardium [38]. Similarly, *NR4A3* is found to be elevated 10 days post-left anterior descending artery ligation ischemia surgery in mice [39]. Overexpression of *NR4A3* significantly reduces infarct size, preventing deterioration of left ventricular function and repression of neutrophil infiltration in the heart of mice after coronary artery ligation and relates to the activation of *JAK2/STAT3* and the inhibition of *STAT3*-dependent *NF-κB* signaling pathways [40]. Additionally, it has to be pointed out that the *NR4A* subgroup, including *NR4A3*, is an immediate early response gene induced by diverse physiological stimuli, i.e., mechanical agitation, calcium, and inflammation cytokines [41]. This reinforces our data that, in very early-stage AMI, *NR4A3* has a significant 16-fold clustering coefficient climb and 42% gene expression increase. Yet, despite these characteristics, implications of the nuclear factor *NR4A3* in CD146^+^-related myocardial disorders remain a mystery.

## The CCL20 hub gene

*CCL20*, a C–C motif chemokine, is a chemoattractant for recruiting leukocytes to sites of injury and inflammation. *CCL20* secretion is induced by pro-inflammatory chemokines and cytokines, such as *CXCL12*, *IL17*, *IL1β*, *IL6*, and is in part related to *JAK/STAT* pathway signaling in multiple cell types [42–44]. *IL6* and soluble *IL6* receptor stimulate *STAT3* binding to the *CCL20* promotor, and *IL17* stimulates the phosphorylated *NF-κB* binding to the *CCL20* promoter in murine astrocytes, facilitate neuroinflammation within the central nervous system [42]. In addition, the co-expression of *CCL20* receptor *CCR6* and CD146 is a marker of effector memory Th17 cells which mediate migration and is thought to be essential for inflammation in human psoriasis [8]. Moreover, it is reported that *CCL20* levels are elevated in the serum of clinical patients with ischemic myocardial infarction [45, 46]. In vitro study shows that *CCL20* expression increases in CD146^+^ human mesenchymal stromal cells at the early pro-inflammatory phase in fracture healing [47]. Thus, we hypothesize that *CCL20* binding its receptor *CCR6* is likely what drives the CD146-mediated vessel inflammation progress in early AMI phase.

## Functional gene clusters

In terms of functional models, DCGs are self-organized into two clusters, the *NR4A3* and *CCL20* clusters, with 18 genes and 12 genes in each cluster, respectively. All genes are directly linked to its hub gene and partly linked to adjacent genes. Protein–protein connection analyzed by STRING database produces a structure that is greatly similar to our network pattern in which *CCL20* connects with *CXCL16*, *IL1R2*, *MMP9*, *NLRP3*, *BCL6 LILRB2*, *PELI1*, *CLEC4E*, *FCER1G*, and *NR4A3* connects with *NR4A2*, *FOSL2*, *RNF144B*, *CDKN1A*, *SOCS3*. A few of the gene–gene correlations within clusters are stated in inflammatory diseases. *MMP9* activation correlates with *CCL20* expression in astrocytes via *Notch-1/Akt/NF-κB* pathway promoting leukocyte migration across the blood–brain barrier [48]. *NR4A2* and *NR4A3* as orphan nuclear receptors mediate neutrophil number and survival in chronic inflammatory signals in multiple hematologic disorders [49–51]. *FOSL2* acts as an activating protein-1 transcription factor promoting hematopoietic progenitor cells transition to macrophages and neutrophils in an *SOCS3*-dependent manner is reported [52]. Nevertheless, most of the cluster functions are rarely reported in AMI pathogenesis.

Taken together, *NR4A3* and *CCL20* clusters are novel functional modules in CD146^+^ cell-mediated immuno­inflammatory balance, triggering increased susceptibility to vascular deterioration and accelerating myocardial injury. Meanwhile, we propose that *NR4A3* and *CCL20* are promising biomarkers for clinical diagnosis and potential therapeutic candidates since they largely impact the early AMI development*.* In-depth studies are necessary for understanding the mechanisms of peripheral CD146^+^ cells in cardiovascular disease.

## Supplementary Information

**Additional file 1: Figure S1.** A CCL20 cluster genes. B NR4A3 cluster genes. **Figure S2.** Protein-protein connection of DCGs by STRING database.

**Additional file 2: Table S1. **Power-law indexes of degree distribution for the control network in the discovery and validation cohorts. **Table S2. **Clustering coefficients of DCGs in the discovery cohort, the validation cohort, and the discovery + validation cohort.

## Data Availability

All data are included in the manuscript.
