# Supplementary material for: NR4A3 and CCL20 clusters dominate the genetic networks in CD146+ blood cells during acute myocardial infarction in humans
Source: Eur J Med Res. 2021 Sep 26;26:113. doi: 10.1186/s40001-021-00586-8 (PMC8474787; doi:10.1186/s40001-021-00586-8)

Supplement Figure 1A.

CCL20 cluster genes

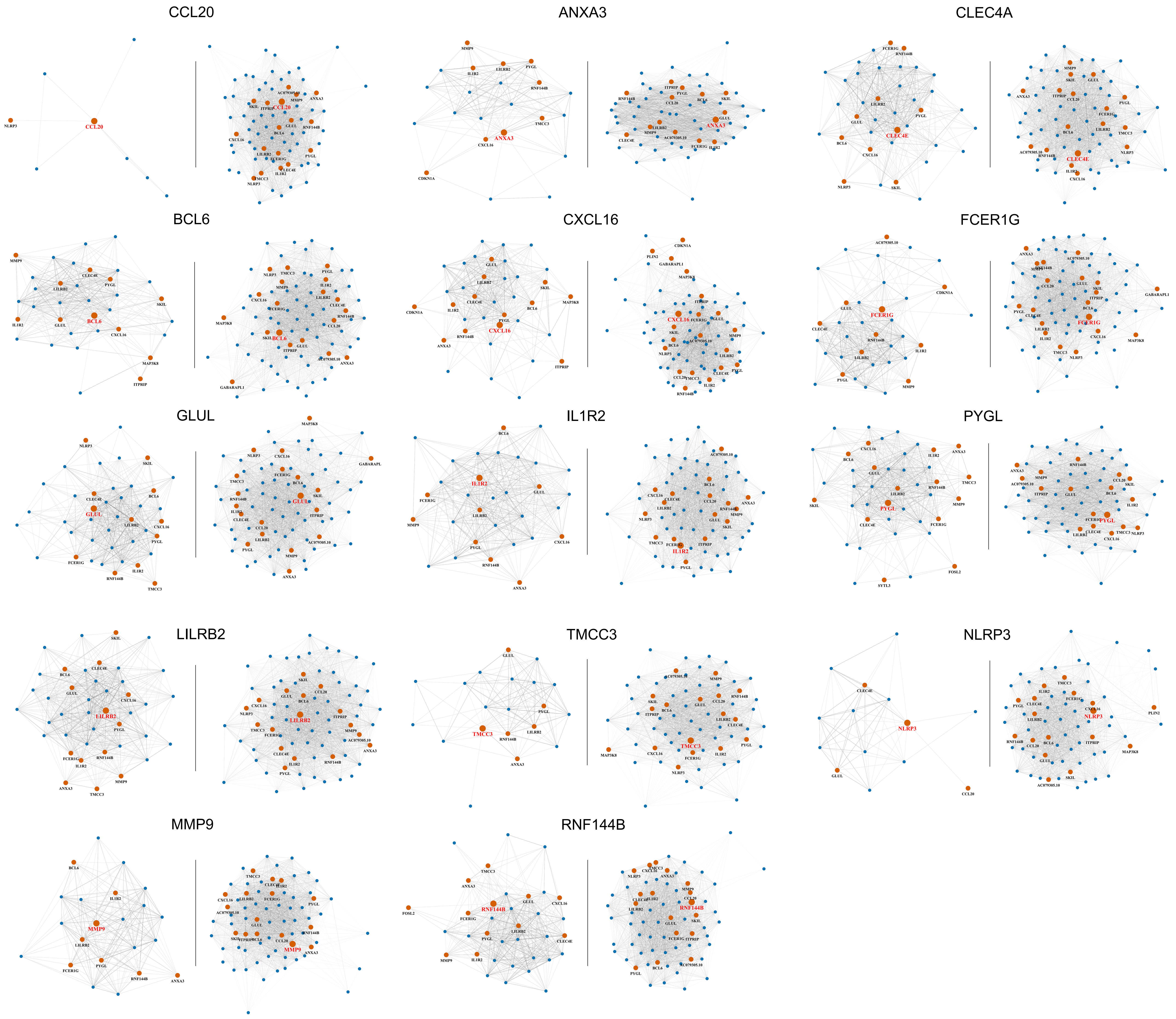

Supplement Figure 1B.

NR4A3 cluster genes

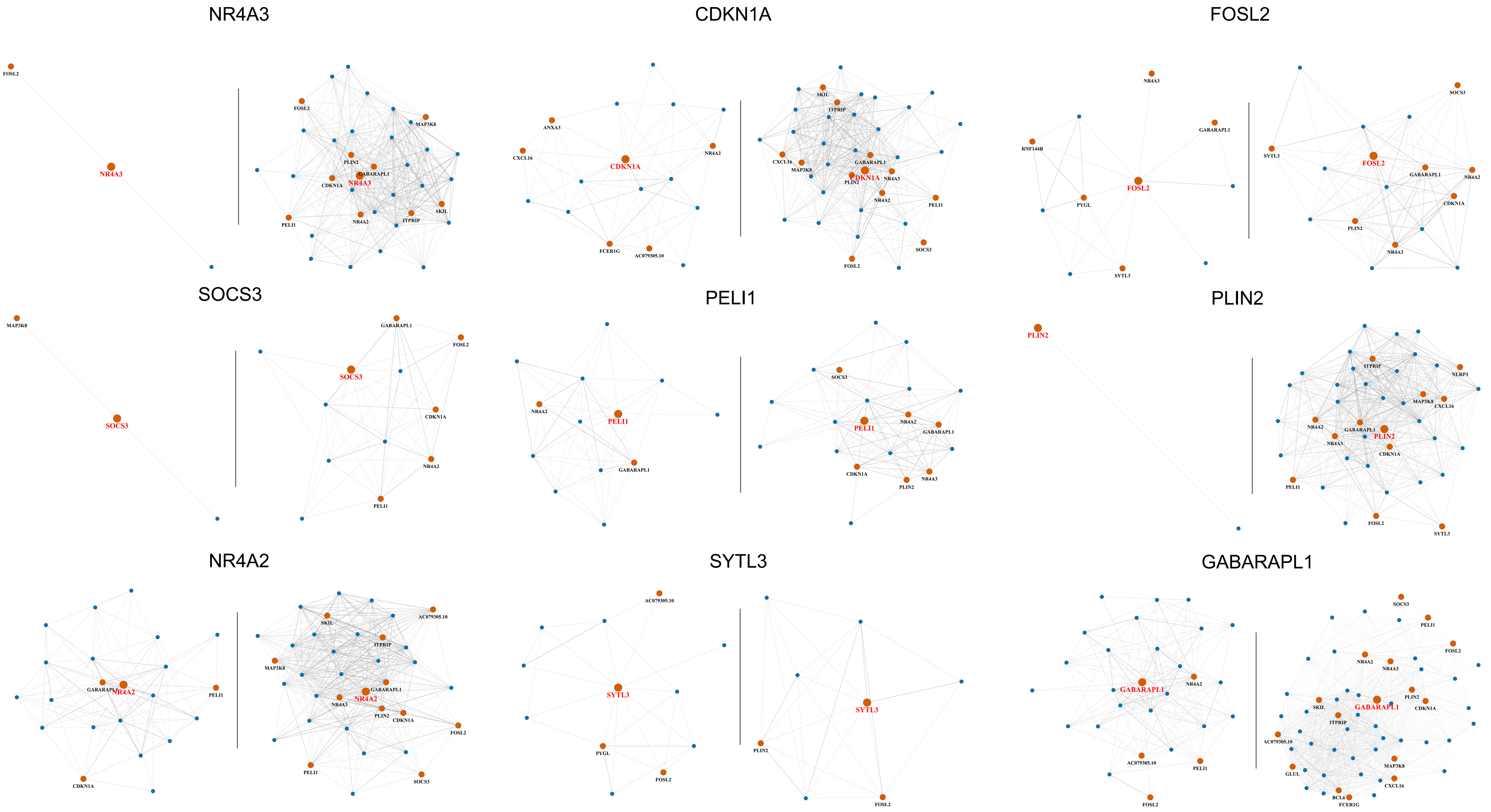

Intermediate genes

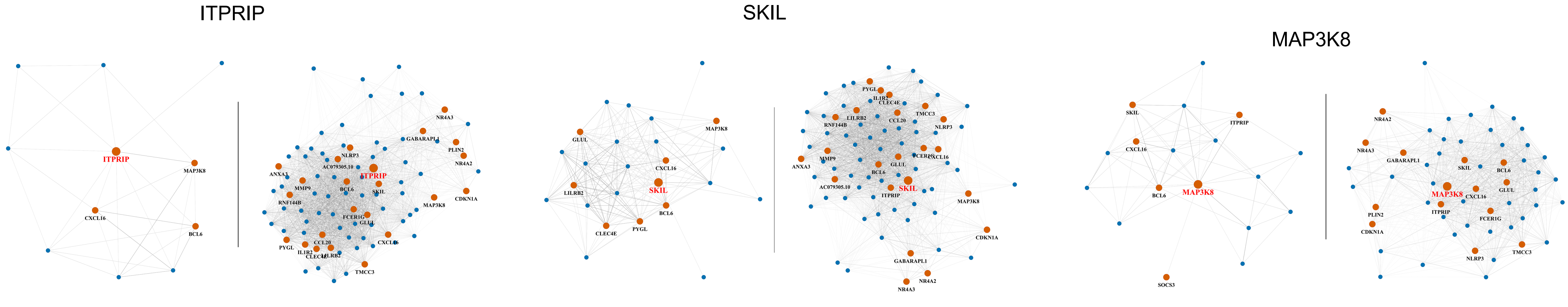

Supplement Figure 2.

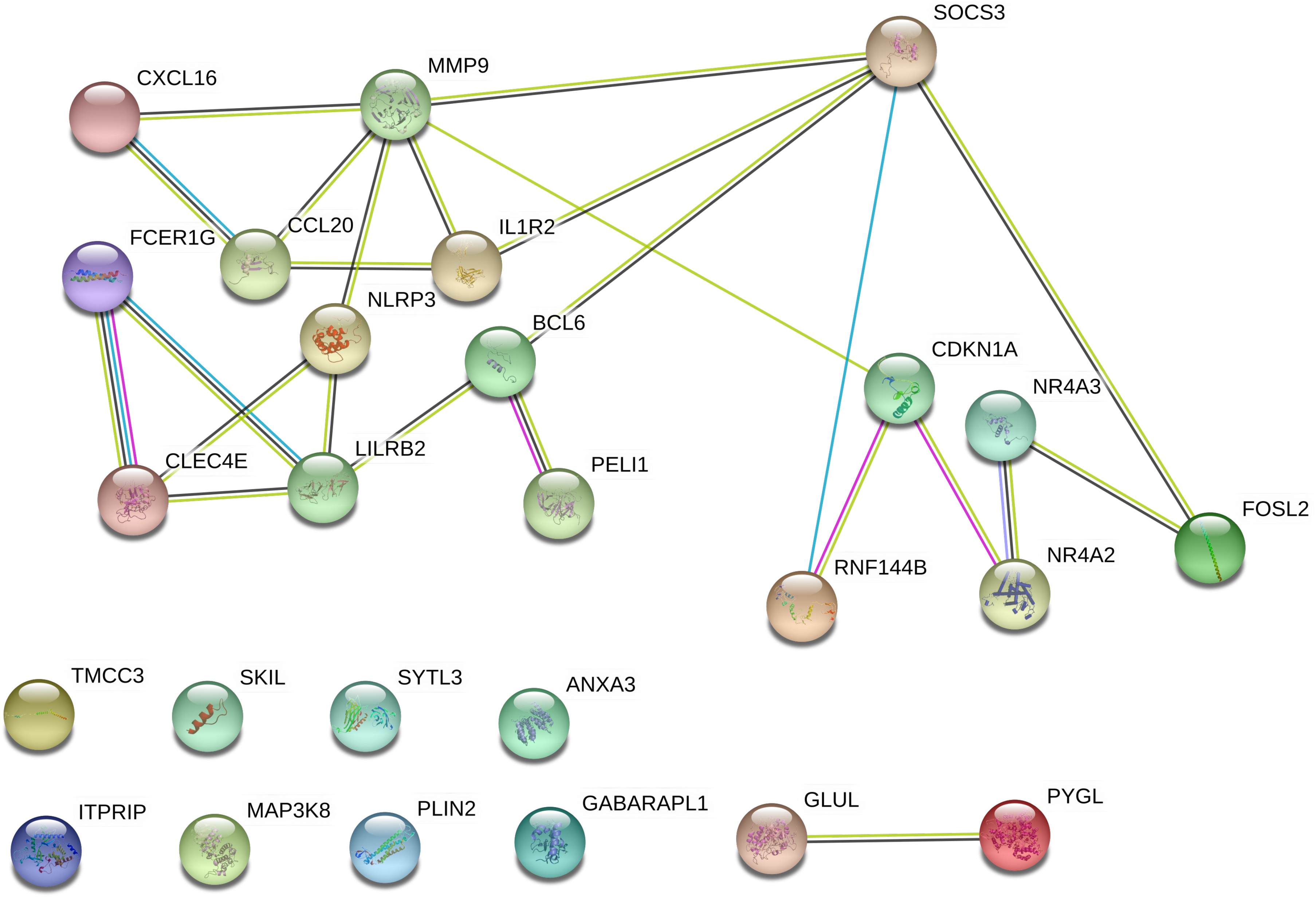

Supplement: Supplementary file 1 — Additional file 1: Figure S1. A CCL20 cluster genes. B NR4A3 cluster genes. Figure S2. Protein-protein connection of DCGs by STRING database. [file 40001_2021_586_MOESM1_ESM.pdf]
