# Supplementary material for: NR4A3 and CCL20 clusters dominate the genetic networks in CD146+ blood cells during acute myocardial infarction in humans
Source: Eur J Med Res. 2021 Sep 26;26:113. doi: 10.1186/s40001-021-00586-8 (PMC8474787; doi:10.1186/s40001-021-00586-8)
Supplement: Supplementary file 2 — Additional file 2: Table S1. Power-law indexes of degree distribution for the control network in the discovery and validation cohorts. Table S2. Clustering coefficients of DCGs in the discovery cohort, the validation cohort, and the discovery + validation cohort. [file 40001_2021_586_MOESM2_ESM.pdf]

Supplement Table 1. Power-law indexes of degree distribution for the control network in the discovery and validation cohorts.

Threshold	Power-law indexes of degree distribution	
	Discovery cohort	Validation cohort
0.1	3.50	3.50
0.2	3.50	3.50
0.3	3.50	3.50
0.4	3.50	1.50
0.5	2.47	1.56
0.6	1.86	3.50
0.7	2.16	3.50
0.8	3.50	3.50
0.9	0.00	3.50

Supplement Table 2. Clustering coefficients of DCGs in the discovery cohort, the validation cohort, and the discovery + validation cohort.

	Discovery cohort			Validation cohort			Discovery + validation cohort		
Genes	Control	AMI	**Δ （AMI-Control）**	Control	AMI	**Δ （AMI-Control）**	Control	AMI	**Δ （AMI-Control）**
CCL20	0.073	0.816	0.744	0.526	0.641	0.115	0.367	0.714	0.347
NR4A3	0.038	0.638	0.600	0.309	0.697	0.388	0.451	0.696	0.245
PLIN2	0.120	0.667	0.547	0.327	0.603	0.276	0.163	0.529	0.366
ANXA3	0.346	0.890	0.545	0.060	0.463	0.403	0.210	0.640	0.430
AC079305.10	0.339	0.814	0.475	0.124	0.508	0.385	0.224	0.656	0.432
TMCC3	0.421	0.896	0.474	0.033	0.604	0.571	0.078	0.636	0.558
FOSL2	0.129	0.555	0.426	0.422	0.676	0.254	0.455	0.610	0.155
ITPRIP	0.198	0.599	0.401	0.267	0.480	0.213	0.414	0.576	0.162
NLRP3	0.384	0.761	0.377	0.191	0.603	0.411	0.215	0.643	0.428
CDKN1A	0.260	0.626	0.366	0.087	0.791	0.705	0.332	0.466	0.134
SKIL	0.335	0.687	0.352	0.260	0.577	0.317	0.237	0.641	0.405
MMP9	0.453	0.793	0.340	0.236	0.355	0.119	0.203	0.627	0.423
MAP3K8	0.246	0.577	0.331	0.128	0.602	0.474	0.366	0.591	0.226
BCL6	0.400	0.728	0.327	0.064	0.602	0.538	0.359	0.569	0.209
NR4A2	0.378	0.668	0.290	0.257	0.761	0.504	0.114	0.587	0.473
FCER1G	0.456	0.745	0.289	0.373	0.619	0.246	0.347	0.605	0.258
RNF144B	0.550	0.839	0.288	0.523	0.642	0.119	0.282	0.753	0.471

Supplement Table 2 (continue). Clustering coefficients of DCGs in the discovery cohort, the validation cohort, and the discovery + validation cohort.

	Discovery cohort			Validation cohort			Discovery + validation cohort		
Genes	Control	AMI	**Δ （AMI-Control）**	Control	AMI	**Δ （AMI-Control）**	Control	AMI	**Δ （AMI-Control）**
IL1R2	0.552	0.840	0.288	0.343	0.706	0.363	0.392	0.745	0.353
PYGL	0.542	0.813	0.270	0.064	0.689	0.625	0.281	0.643	0.363
SYTL3	0.130	0.388	0.258	0.000	0.278	0.278	0.094	0.282	0.187
LILRB2	0.553	0.792	0.239	0.033	0.680	0.647	0.237	0.612	0.375
SOCS3	0.040	0.254	0.214	0.472	0.908	0.435	0.333	0.692	0.359
PELI1	0.324	0.482	0.158	0.581	0.746	0.165	0.515	0.743	0.228
GABARAPL1	0.363	0.490	0.128	0.493	0.651	0.158	0.319	0.670	0.351
CXCL16	0.466	0.589	0.123	0.443	0.731	0.288	0.222	0.625	0.403
GLUL	0.582	0.697	0.115	0.584	0.739	0.155	0.361	0.609	0.248
CLEC4E	0.707	0.815	0.108	0.328	0.625	0.297	0.255	0.635	0.380
